# Inequalities in health-related quality of life according to age, gender, educational level, social class, body mass index and chronic diseases using the Spanish value set for Euroquol 5D-5L questionnaire

**DOI:** 10.1186/s12955-019-1134-9

**Published:** 2019-04-18

**Authors:** Arantzazu Arrospide, Mónica Machón, Juan M. Ramos-Goñi, Oliver Ibarrondo, Javier Mar

**Affiliations:** 1 Unidad de Investigación AP-OSIs Gipuzkoa, Hospital Alto Deba, Avda Navarra 16, Arrasate-Mondragón, 20500 Gipuzkoa Spain; 2 Red de Investigación en Servicios de Salud en Enfermedades Crónicas (REDISSEC), Bilbao, Spain; 3 Instituto de Investigación Sanitaria Biodonostia, San Sebastián, Spain; 4 Unidad de Investigación AP-OSIs Gipuzkoa, Osakidetza, San Sebastián, Spain; 5 Centro de Investigación en Cronicidad Kronikgune, Barakaldo, Spain; 6 Axentiva Solutions, Tacoronte, Spain

**Keywords:** Health-related quality of life, EQ-5D-5L, Body mass index, Chronic conditions, Social class

## Abstract

**Background:**

Reducing health inequalities on the basis of social factors has been a key driver in the development of Public Health policies. Health-related quality of life is a global indicator useful to assess health inequalities within a society. The objective of this study was to identify inequalities on health by analysing the interactive effects of gender, age, educational level, social class, body mass index and chronic diseases on health-related quality of life in a Spanish population sample.

**Methods:**

We used data from the Spanish National Health Survey 2011–2012. Health-related quality of life was measured by the EQ-5D-5L instrument applying the Spanish value set. Probability of being in perfect health was ascertained by logistic regression models including gender, age, educational level, body mass index and social class and the corresponding terms of interaction. A two-part model combining logistic regression analysis and generalized linear models was applied to calculate the adjusted utility loss associated with chronic conditions (disutility values).

**Results:**

The sample used for analysis contained 18,450 individuals. The mean age was 50 years, 51.3% were women, 55% were overweight or obese and 46.7% had low social status. The mean utility was 0.94 in men and 0.89 in women. Elderly women, obese people, those of low social class and those with chronic conditions had significant lower utility values. Within the regression analysis, interaction assessment revealed that the detrimental effect of obesity disappeared in higher social classes. Utility values for all chronic conditions considered were lower in women than in men and were on a gradient within social class, the lowest for individuals declaring stroke. The greatest decrease on health-related quality of life was determined by declaration of stroke (17.6%) or mental diseases (18.6%).

**Conclusions:**

The interactive effects of gender, age, educational level, social class, body mass index and chronic diseases on health-related quality of life in the Spanish population revealed important inequalities in health. Social class acted as a modulator of the stigma associated with obesity. Chronic conditions producing loss of autonomy had the greatest impact on reduction of health-related quality of life. This is the first study using the Spanish EQ-5D-5L value set to estimate utilities.

**Electronic supplementary material:**

The online version of this article (10.1186/s12955-019-1134-9) contains supplementary material, which is available to authorized users.

## Background

Health inequalities within countries are mainly associated with a gradient in social factors that could influence, for example, disease onset and response to treatment [1]. Reduction of health inequalities based on ethnicity, social class, and other social factors has been a key driver in the development of public health policies designed to break the link between poverty and disease [2, 3]. Health-related quality of life (HRQL), as an indicator collected in population health surveys, can be used to monitor self-reported health differences [4]. Among the instruments for assessing HRQL available in the literature, the generic EQ-5D questionnaire is one of the most frequently used to estimate utility values [5]. Two versions have been developed for use in the adult population: the EQ-5D-3 L and the EQ-5D-5L [6], which was structured to overcome the lack of sensitivity observed in the three-level version [7]. With the EQ-5D instrument, health utilities for many countries can be obtained and employed to calculate quality adjusted life years (QALY) [8]. The value set to calculate preferences from EQ-5D-5L was developed in Spain, and, therefore, Spanish population values can be obtained [9, 10].

The EQ-5D-5L was incorporated in the Spanish National Health Survey 2011–2012, allowing the calculation of utility values for the Spanish general population according to different characteristics [10, 11]. Results from previous research that assessed the HRQL via the EQ-5D [12–15] showed that women, people of low social class, obese people and those with chronic diseases usually reported lower HRQL, but the research did not address the interaction among these factors to identify the origin of those self-reported health differences [2].

The objective of this study was to identify drivers of inequalities on health by analysing the interactive effects of gender, age, educational level, social class, body mass index (BMI) and chronic diseases on HRQL in a representative sample of the Spanish population using the Spanish value set for EQ-5D-5L.

## Methods

### Study design and study population

This was a cross-sectional study based on data from the Spanish Health Survey (SHS), performed by the Spanish Ministry of Health, Social Services and Equity [11]. The SHS is a nation-wide survey conducted periodically in a representative sample of non-institutionalized Spanish population aged 15 years or older. Individuals were selected via a multistage sampling, with the first-stage units being census sections, the second-stage units main family dwellings and the final units individuals; the individuals were randomly chosen from the family relatives accessible at the time the interview was carried out. This survey was carried out in Spanish; however, in the case of individuals that were not able to answer the questionnaire in Spanish, a relative was allowed to help translate in order to keep the sample representative. The data collection period was between June 2011 and June 2012. Interviews were conducted by trained personnel in the randomly selected households through computer-assisted personal interview. The survey data were downloaded from the Spanish Statistical Office website, where a more detailed description can be found.

### Measures

The following variables were considered as covariates in the analysis: gender, age, BMI, chronic conditions, educational level and social class. Social class, following the definition by the Health Determinants Taskforce of the Spanish Epidemiology Society [16], has six categories, which were grouped into three in this analysis: high (class I-II: company directors, athletes and artists), middle (class III-IV: intermediate managers, self-employed workers, supervisors and technically qualified workers) and low social class (class V-VI: farmers, other semi-skilled workers, unskilled workers). Educational level was also included in three different categories: high (professional or university level studies), middle (compulsory studies), low education level (less than compulsory studies). HRQL was measured by use of the EQ-5D-5L instrument [7, 9], which was included in the Spanish Health Survey adult questionnaire.

This consisted of five dimensions: mobility, self-care, usual activities, pain/discomfort, and anxiety/depression. Each dimension was measured on five response levels: no problem, slight, moderate, severe and inability/extreme problems. A total of 3215 possible health states could be observed, ranging from 11,111 (excellent health state) to 55,555 (worst health status). These health states were converted into a utility index through the Spanish Value Set algorithm in Stata (provided in the appendix of their paper) by Ramos-Goñi et al. [9].

The BMI was obtained from self-reported height and weight. It was calculated as weight (in kilograms) divided by the square of the height (in metres) and individuals were classified, based on World Health Organization guidelines [17], as normal weight (< 25 kg/m^2^), overweight (25–29.9 kg/m^2^) or obese (≥30 kg/m^2^). Participants were asked about having received a diagnosis of any chronic conditions in the last 12 months: acute myocardial infarction (AMI); other heart diseases; arthrosis, arthritis or rheumatism; chronic bronchitis, emphysema, chronic obstructive pulmonary disease (COPD); diabetes; cirrhosis, hepatic dysfunction; mental health problems (such as anxiety and depression) or stroke.

### Statistical analysis

Statistical differences between mean utility values for overweight and obese people compared to those of normal weight were assessed using the Student t-test. Logistic regression analysis was applied to estimate the probability of being in perfect health (utility value equal to 1) and having answered “1” (no problem) in each dimension of EQ-5D-5L (mobility, self-care, usual activities, pain/discomfort and anxiety/ depression). In the multivariate regression, interactions between age and gender, gender and BMI categories, as well as between social class and BMI categories, were included.

In order to assess adjusted loss of HRQL due to chronic diseases, two-step regression analysis was carried out [18]. First, logistic regression allowed estimating the adjusted probability (*p*) of being in perfect health status in each subgroup. Second, generalized linear models were used to estimate mean utility values (*v*) in the population not in perfect health (utilities lower than 1), both for those with and without chronic disease. Mean utility value for each of the analysed subgroups was calculated using the following formula: u = p + (1 − p) ∙ v. An adjusted disutility value for mean age (50 years) was calculated as the difference between the estimated mean utilities for subgroups with and without chronic disease. The percentage of adjusted disutility loss was calculated by taking as reference the utility value of the correspondent population without chronic diseases. The two-step regression analysis was carried out twice, first with the gender variable and second by its interaction with the disease diagnosis, in order to separately estimate the effect for each subpopulation. The analysis was repeated using the social class variable instead of gender.

## Results

We used 18,450 individuals in the analysis (Table 1), after excluding 2557 individuals due to missing responses in: EQ-5D-5L (*n* = 28), height or weight (*n* = 1927) or social class (*n* = 602). The mean age of our sample was 50.0 (SD 18.2) years, and 51.3% were women. In addition, 46.7% had a low social class, 23.3% low educational level and the prevalence of the chronic diseases varied from 1.1% (cirrhosis, hepatic dysfunction) to 35.5% (arthrosis, arthritis and rheumatism). In relation to the BMI, 45.0% were normal weight (BMI < 25 kg/m^2^), 37.6% overweight (BMI 25–30 kg/m^2^) and 17.4% obese (BMI > 30 kg/m^2^).

**Table 1:** Main characteristics of the studied sample

Variables	Men		Women		Total	
	N	%	N	%	N	%
Total	8983	48.7%	9467	51.3%	18450	100.0%
Age						
Mean, (SD)	48.9	17.7	51.0	18.7	50.0	18.2
< 30	1281	14.3%	1243	13.1%	2524	13.7%
30–39	1749	19.5%	1707	18.0%	3456	18.7%
40–49	1829	20.4%	1766	18.7%	3595	19.5%
50–59	1521	16.9%	1543	16.3%	3064	16.6%
60–69	1293	14.4%	1369	14.5%	2662	14.4%
70–79	850	9.4%	1148	12.1%	1998	10.8%
80–89	425	4.7%	588	6.2%	1013	5.5%
≥ 90	35	0.4%	103	1.1%	138	0.8%
Education level						
Low	1956	21.8%	2347	24.8%	4303	23.3%
Middle	4155	46.2%	4127	43.6%	8282	44.9%
High	2872	32.0%	2993	31.6%	5865	31.8%
Social class						
Low	4122	45.9%	4498	47.5%	8620	46.7%
Middle	3138	34.9%	3146	33.2%	6284	34.1%
High	1723	19.2%	1823	19.3%	3546	19.2%
BMI						
< 25	3204	35.7%	5103	53.9%	8307	45.0%
25–30	4129	46.0%	2813	29.7%	6942	37.6%
> 30	1650	18.3%	1551	16.4%	3201	17.4%
Chronic conditions diagnosed						
Acute myocardial infarction	287	3.2%	108	1.1%	395	2.1%
Other heart diseases	606	6.8%	629	6.6%	1235	6.7%
Arthrosis, arthritis or rheumatism	2443	27.2%	4115	43.5%	6558	35.5%
Chronic bronchitis, emphysema, COPD	488	5.4%	466	4.9%	954	5,2%
Diabetes	764	8.5%	716	7.6%	1480	8.0%
Cirrhosis, hepatic dysfunction	111	1.2%	98	1.0%	209	1.1%
Mental health problems	662	7.4%	1651	17.4%	2313	12.5%
Stroke	136	1.5%	112	1.2%	248	1.3%
Number of chronic conditions diagnosed						
None	5393	60.0%	4466	47.2%	9859	53.5%
One or two	2313	25.8%	3008	31.8%	5321	28.8%
More than two	1277	14.2%	1993	21.0%	3270	17.7%

*SD* standard deviation, *BMI* body mass index, *COPD* chronic obstructive pulmonary disease

Additional file 1 Table S1 (available in the supplementary file) shows that by gender, utility values for men were higher compared to those for women in all BMI and social class categories. Considering BMI, mean utilities were significantly lower in obese people compared to those of normal weight in both men and women and social class levels. Women in the middle and high social classes showed a significantly lower utility compared to normal-weight women.

**Fig. 1:**
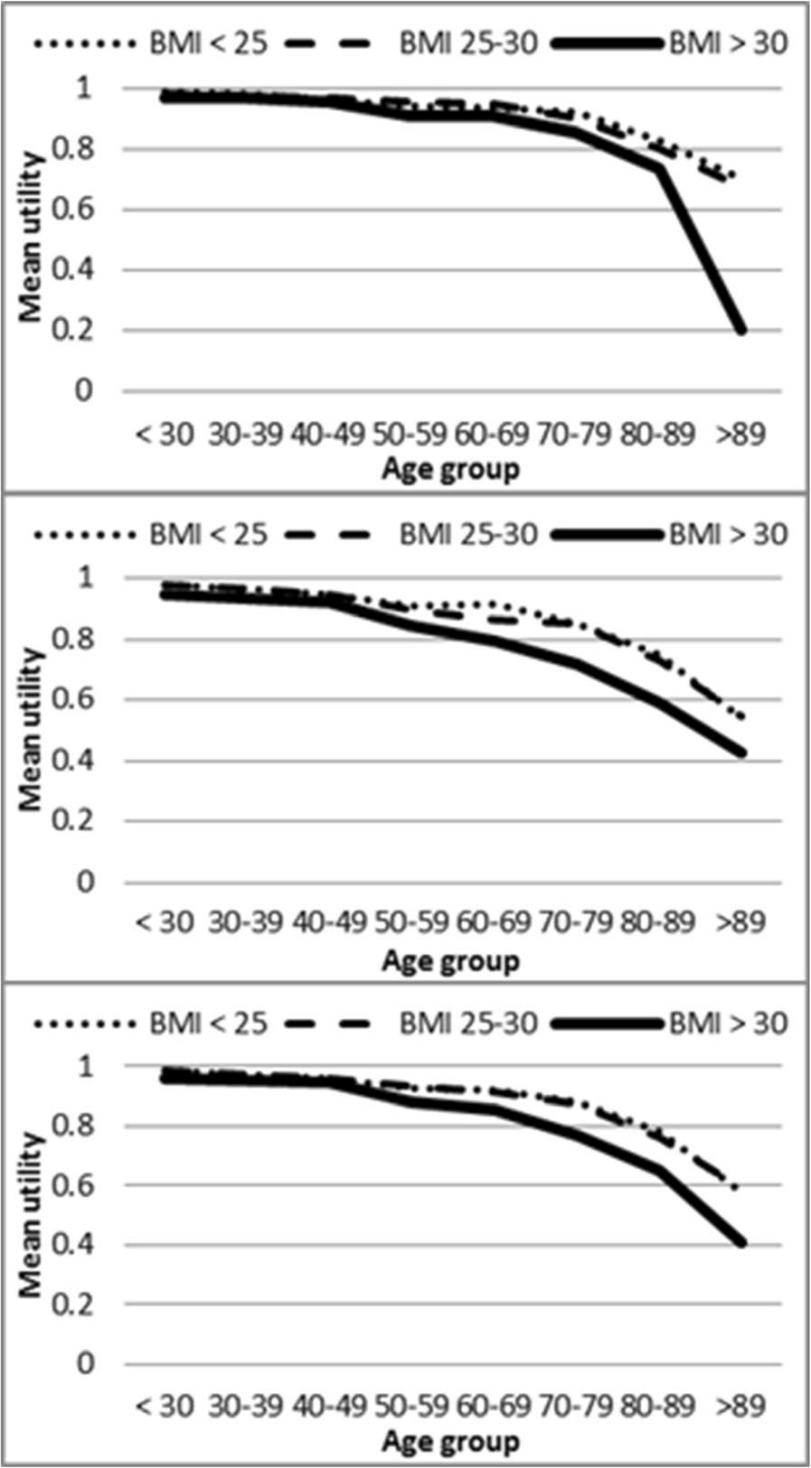
**a** Mean utilities by age groups and body mass index categories in men. **b** Mean utilities by age groups and body mass index categories in women. **c** Mean utilities by age groups and body mass index categories in total population

**Fig. 2:**
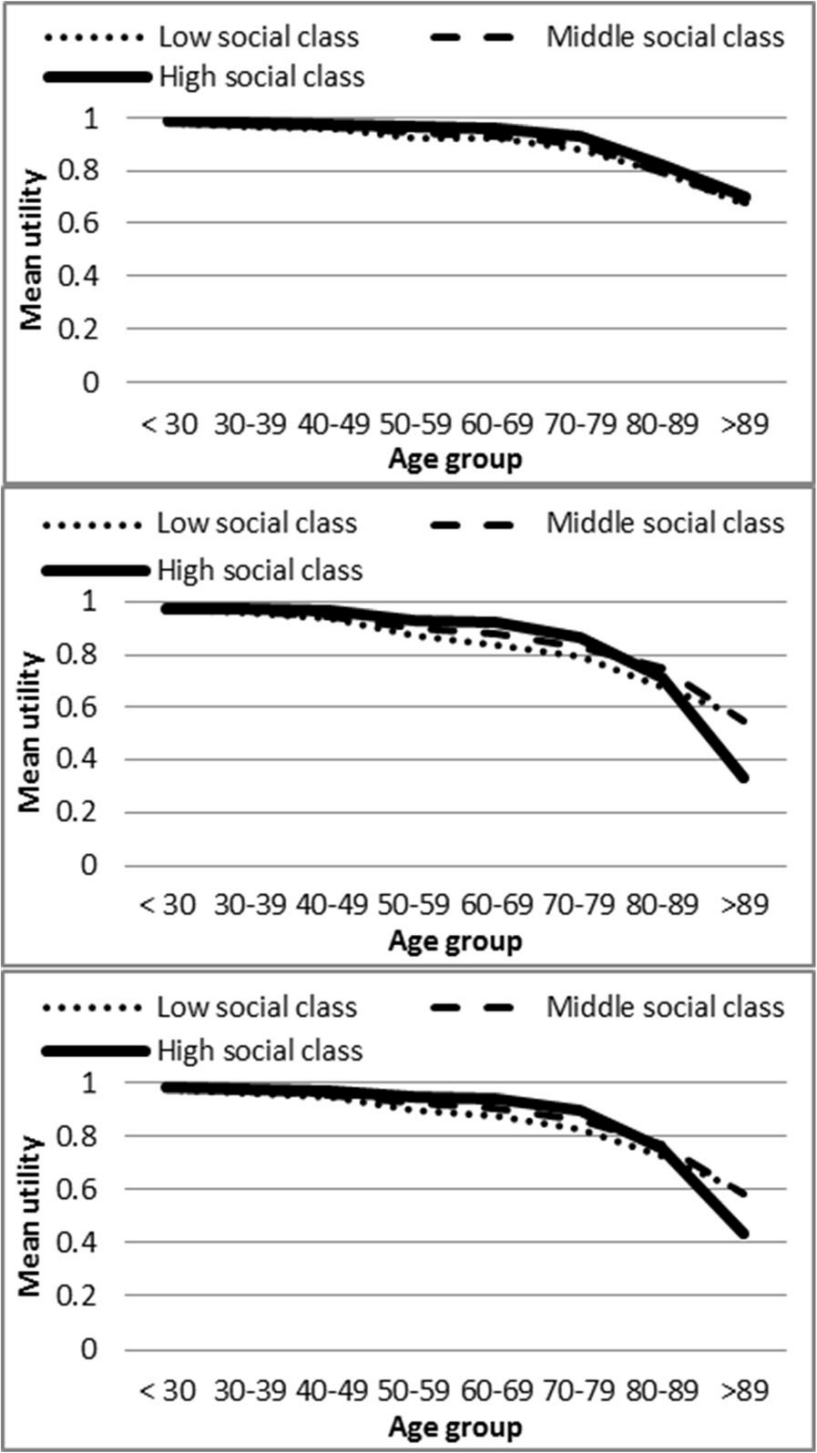
**a** Mean utilities by age groups and social class in men. **b** Mean utilities by age groups and social class in women. **c** Mean utilities by age groups and social class in total population

Utilities decreased as age increased in the three BMI categories and in both men and women (Fig. 1 and Additional file 1: Table S2). The gap was especially prominent between overweight and obese people, as curves for normal weight and overweight overlapped in many age groups. The same effect of ageing is apparent in Fig. 2 and Additional file 1: Table S3, which describes mean utilities by age groups and social class. In Additional file 1: Table S4 the number of chronic conditions was disaggregated by social class and BMI categories, showing a gradient both for social class and for BMI categories in the percentage of individuals without chronic conditions.

Table 2 shows in the first column the results of the logistic analysis estimating the adjusted probability of being in perfect health. The other columns contain the results of the same procedure using as the dependent variable the answer “1” (no problem) in each dimension of EQ-5D-5L. The analysis found that for each year of increased age, the odds ratio (OR) decreased significantly less for individuals younger than 70 years (OR: 0.96) than for those older than 70 (OR: 0.96*0.97 = 0.93). Women, overall, were less likely to report perfect health compared to men (OR: 0.60, 95%, confidence intervals [CI]: 0.53–0.67). The effect associated with age was also slightly greater for women (OR: 0.99, 95% CI: 0.99–1.00).

**Table 2:** Probability of being in perfect health status (utility equal to 1) and having one in each dimension of EQ-5D-5L (mobility, selfcare, usual activities, pain/discomfort and anxiety depression)

Logistic regression	Utility	Mobility	Selfcare	Usual activities	Pain/discomfort	Anxiety & Depression
	OR (95 CI)	OR (95 CI)	OR (95 CI)	OR (95 CI)	OR (95 CI)	OR (95 CI)
Age	0.96 (0.96, 0.97)**	0.94 (0.94, 0.95)**	0.95 (0.94, 0.96)**	0.95 (0.95, 0.96)**	0.96 (0.96, 0.97)**	0.97 (0.97, 0.98)**
Age ≤ 70 years	1	1	1	1	1	1
Age > 70 years	2.26 (1.52, 3.36)**	2.50 (1.68, 3.72)**	4.38 (2.70, 7.12)**	4.03 (2.65, 6.13)**	1.29 (0.89, 1.86)	1.23 (0.82, 1.87)
Age > 70 years *Age	0.97 (0.95, 0.98)**	0.96 (0.95, 0.98)**	0.94 (0.92, 0.96)**	0.94 (0.93, 0.96)**	0.99 (0.98, 1.00)	1.01 (1.00, 1.03)
Men	1	1	1	1	1	1
Women	0.60 (0.53, 0.67)**	1.02 (0.84, 1.23)	0.78 (0.57, 1.06)	0.85 (0.69, 1.04)	0.57 (0.50, 0.64)**	0.58 (0.50, 0.67)**
Women * Age	0.99 (0.99, 1.00)*	0.99 (0.99, 1.00)*	0.99 (0.98, 1.00)	0.99 (0.98, 1.00)**	1.00 (0.99, 1.00)*	0.99 (0.99, 1.00)*
Low education level	1	1	1	1	1	1
Middle education level	1.15 (1.05, 1.26)**	1.13 (1.01, 1.27)*	1.33 (1.13, 1.56)**	1.20 (1.06, 1.36)**	1.11 (1.01, 1.22)*	1.15 (1.04, 1.29)*
High education level	1.44 (1.29, 1.61)**	1.72 (1.46, 2.04)**	1.73 (1.33, 2.24)**	1.64 (1.36, 1.97)**	1.38 (1.22, 1.55)**	1.50 (1.30, 1.72)**
Low social class	1	1	1	1	1	1
Middle social class	1.37 (1.22, 1.55)**	1.45 (1.20, 1.76)**	1.48 (1.12, 1.96)**	1.40 (1.14, 1.72)**	1.29 (1.13, 1.48)**	1.38 (1.18, 1.60)**
High social class	1.25 (1.46, 1.66)**	1.79 (1.40, 2.31)**	1.59 (1.10, 2.29)*	1.94 (1.47, 2.55)**	1.46 (1.25, 1.72)**	1.40 (1.16, 1.68)**
BMI < 25	1	1	1	1	1	1
BMI 25–30	1.07 (0.93, 1.23)	1.20 (0.98, 1.48)	1.08 (0.80, 1.47)	1.24 (0.99, 1.56)	0.97 (0.83, 1.14)	1.26 (1.04, 1.52)*
BMI > 30	0.71 (0.60, 0.83)**	0.66 (0.53, 0.82)**	0.70 (0.50, 0.98)*	0.78 (0.61, 0.99)**	0.70 (0.59, 0.84)**	0.95 (0.77, 1.18)
BMI 25–30 * Women	0.82 (0.70, 0.96)*	0.67 (0.53, 0.85)**	1.00 (0.71, 1.40)	0.74 (0.57, 0.95)*	0.88 (0.74, 1.05)	0.72 (0.59, 0.88)**
BMI > 30 * Women	0.76 (0.62, 0.91)**	0.61 (0.48, 0.79)**	0.66 (0.46, 0.95)*	0.59 (0.45, 0.78)**	0.73 (0.59, 0.89)**	0.67 (0.53, 0.85)**
BMI 25–30 * Middle social class	0.84 (0.71, 0.99)*	0.85 (0.66,1.09)	0.77 (0.53, 1.11)	0.93 (0.71, 1.22)	0.90 (0.75, 1.08)	0.94 (0.76, 1.17)
BMI 25–30 * High social class	1.00 (0.81, 1.23)	0.69 (0.49, 0.95)*	0.76 (0.46, 1.24)	0.63 (0.44, 0.90)*	1.04 (0.83, 1.31)	0.99 (0.76, 1.30)
BMI > 30 * Middle social class	0.75 (0.61, 0.92)**	0.73 (0.55, 0.96)*	0.82 (0.55, 1.22)	0.85 (0.63, 1.14)	0.85 (0.69, 1.06)	0.85 (0.66, 1.09)
BMI > 30 * High social class	1.16 (0.87, 1.54)	0.81 (0.54, 1.21)	0.98 (0.55, 1.75)	0.73 (0.47, 1.14)	1.19 (0.87, 1.61)	0.78 (0.56, 1.10)
Constant	2.20 (1.94, 2.49)**	8.67 (7.21, 10.43)**	31.43 (23.51, 42.00)**	11.20 (9.17, 13.68)**	3.87 (3.37, 4.44)**	5.73 (4.89, 6.71)**

*CI* confidence intervals; *p < 0.05; **p < 0.01; *BMI* body mass index

The interaction between BMI and social class showed different results according to social class. As expected, people of normal weight from middle and high social classes were more likely to report perfect health (OR: 1.37, 95% CI: 1.22–1.55; OR: 1.25, 95% CI: 1.46–1.66, respectively) compared to those of low social class. Considering BMI, obese people showed a lower probability of being in perfect health status compared to normal-weight people in low (OR: 0.71, 95% CI: 0.52–0.67) and middle (OR: 0.74, 95% CI: 0.61, 0.91) social classes, but no significant differences between BMI categories were observed for those in the highest social class. In addition, obese women compared to normal-weight men showed a significantly lower probability of being in perfect health (OR: 0.76, 95% CI: 0.62–0.91).

Each dimension of EQ-5D-5L had different probabilities of being equal to “1” according to age, gender, educational level, social class and BMI (Table 2). In the interactions analysis, social class and obesity showed a gradient with lower probability of perfect health in the low social class and in obese groups. The OR related to obesity is significant for all dimensions in low social class, except for anxiety and depression, whereas this significance is maintained only for mobility in the middle social class. In addition, overweight people in the high social class showed significantly worse performance in mobility and usual activities. However, no significant inequalities were observed for the obese groups in high social class in any EQ-5D dimension. Interaction between obesity and gender in women showed a decreased likelihood for no problems in all dimensions of the HRQL questionnaire.

Finally, Table 3 contains the mean utility values observed in people reporting chronic conditions and the corresponding age- and gender-adjusted disutilities (difference between mean utility values for those with and without the chronic disease and average age) in absolute and relative terms. Utility values for all chronic conditions were lower in women than in men. The lowest utility value was obtained for both male and female individuals declaring stroke (men, 0.72 and women, 0.58). Stroke and mental diseases were the chronic diseases that were related to the greatest differences in HRQL (18.3 and 17.0%, respectively). Along the same lines, Table 4 shows disease-specific disutilities for each social class adjusted by age. Those chronic conditions with lowest relative differences in the total population, such as acute myocardial infarction or diabetes, showed the main inequalities when analysed by social class. However, stroke and mental health preserved the main results for the three social classes.

**Table 3:** Disutilities in populations with specific diseases adjusted by gender and age

	Utility^a^	Adjusted disutility		
	Mean (SD)	Estimated Mean^b^	Absolute difference	Relative difference
Men population				
Acute myocardial infarction	0.8249 (0.25)	0.9127	− 0.0413	−4.33%
Other heart diseases	0.8335 (0.22)	0.9019	−0.0534	−5.59%
Arthrosis, arthritis or rheumatism	0.8762 (0.19)	0.9030	−0.0664	−6.85%
Chronic obstructive pulmonary disease	0.8355 (0.22)	0.8987	− 0.0569	−5.95%
Diabetes	0.8576 (0.21)	0.9178	−0.0378	−3.96%
Cirrhosis, hepatic dysfunction	0.8011 (0.26)	0.8477	−0.1062	−11.13%
Mental health diseases	0.7710 (0.25)	0.8051	−0.1587	−16.47%
Stroke	0.7166 (0.30)	0.8074	−0.1471	−15.41%
Women population				
Acute myocardial infarction	0.6591 (0.29)	0.7781	- 0.1413	−15.37%
Other heart diseases	0.7117 (0.28)	0.8294	- 0.0936	−10.14%
Arthrosis, arthritis or rheumatism	0.8153 (0.23)	0.8591	- 0.1025	−10.66%
Chronic obstructive pulmonary disease	0.7478 (0.26)	0.8228	- 0.0998	−10.82%
Diabetes	0.7445 (0.28)	0.8485	- 0.0747	−8.09%
Cirrhosis, hepatic dysfunction	0.7122 (0.27)	0.7993	- 0.1203	−13.08%
Mental health diseases	0.7281 (0.26)	0.7871	- 0.1583	−16.74%
Stroke	0.5814 (0.35)	0.7080	- 0.2119	−23.04%
Total population				
Acute myocardial infarction	0.7796 (0.27)	0.8836	− 0.0527	−5.63%
Other heart diseases	0.7714 (0.26)	0.8678	−0.0712	−7.58%
Arthrosis, arthritis or rheumatism	0.8380 (0.22)	0.8760	− 0.0900	−9.32%
Chronic obstructive pulmonary disease	0.7926 (0.24)	0.8641	−0.0747	−7.96%
Diabetes	0.8029 (0.25)	0.8874	−0.0518	−5.52%
Cirrhosis, hepatic dysfunction	0.7594 (0.27)	0.8273	−0.1092	−11.66%
Mental health diseases	0.7403 (0.26)	0.7927	−0.1624	−17.00%
Stroke	0.6556 (0.33)	0.7654	−0.1716	−18.31%

^a^Utility in the subgroup with the chronic condition without adjustment. ^b^Two-step adjusted utility in the subgroup with the chronic condition

**Table 4:** Disutilities in populations with specific diseases adjusted by social class and age

	Utility	Adjusted disutility*		
	Mean (SD)^a^	Estimated Mean^b^	Absolute difference	Relative difference
Low social class				
Acute myocardial infarction	0.7539 (0.26)	0.8631	−0.0654	−7.04%
Other heart diseases	0.7517 (0.26)	0.8598	−0.0713	−7.65%
Arthrosis, arthritis or rheumatism	0.8167 (0.23)	0.8680	−0.0953	−9.89%
Chronic obstructive pulmonary disease	0.7706 (0.25)	0.8508	−0.0811	−8.70%
Diabetes	0.7763 (0.26)	0.8749	−0.0574	−6.16%
Cirrhosis, hepatic dysfunction	0.7360 (0.26)	0.8149	−0.1137	−12.24%
Mental health diseases	0.7176 (0.27)	0.7762	−0.1765	−18.52%
Stroke	0.6582 (0.32)	0.7980	−0.1305	−14.05%
Middle social class				
Acute myocardial infarction	0.8040 (0.26)	0.9087	−0.0365	−3.86%
Other heart diseases	0.7948 (0.25)	0.8916	−0.0558	−5.89%
Arthrosis, arthritis or rheumatism	0.8533 (0.20)	0.8919	−0.0776	−8.01%
Chronic obstructive pulmonary disease	0.8171 (0.23)	0.8905	−0.0562	−5.93%
Diabetes	0.8309 (0.23)	0.9109	−0.0359	−3.80%
Cirrhosis, hepatic dysfunction	0.7472 (0.29)	0.8293	−0.1165	−12.32%
Mental health diseases	0.7643 (0.24)	0.8162	−0.1445	−15.05%
Stroke	0.6370 (0.35)	0.7677	−0.1786	−18.87%
High social class				
Acute myocardial infarction	0.8271 (0.32)	0.9374	−0.0204	−2.13%
Other heart diseases	0.7960 (0.27)	0.8937	−0.0669	−6.96%
Arthrosis, arthritis or rheumatism	0.8785 (0.20)	0.9089	−0.0671	−6.88%
Chronic obstructive pulmonary disease	0.8448 (0.23)	0.9069	−0.0519	−5.42%
Diabetes	0.8604 (0.24)	0.9305	−0.0281	−2.93%
Cirrhosis, hepatic dysfunction	0.8459 (0.23)	0.9040	−0.0539	−5.63%
Mental health diseases	0.7799 (0.24)	0.8149	−0.1560	−16.07%
Stroke	0.6798 (0.35)	0.8162	−0.1428	−14.89%

^a^Utility in the subgroup with the chronic condition without adjustment. ^b^Two-step adjusted utility in the subgroup with the chronic condition

*Adjusted by gender and age

## Discussion

This study of the interactive effect of social class, BMI and chronic diseases, adjusted by age, gender and educational level, reveals important inequalities in HRQL in the Spanish population. Moreover, our subgroup analysis shows that fewer differences between obese people and those of normal weight were associated with high social class and, thus, highlight the role of higher socioeconomic status as a moderator of health inequalities. It was already known that individuals’ socioeconomic status determines their HRQL. But, the selective effect of obesity supports the prevention of obesity within the policies designed to reduce health inequalities in the Spanish population. This statement could also be applicable to other European countries with similar socioeconomic levels.

As expected, the elderly, women, those with a low educational level, those of low social class, the obese and people with chronic conditions reported significantly lower utility values. Although these findings are not new in the literature [12, 13, 19], our study presents them for the first time based on the values obtained by the application of elicited preferences from the Spanish population using the EQ-5D-5L instrument [9, 10]. Therefore, our results will potentially support future work requiring the use of Spanish utilities in economic evaluation. Although the EQ-5D is the most frequently used instrument for calculating quality-adjusted life years, the EQ-5D-3 L validity was hampered by a ceiling effect [20]. EQ-5D-5L represents an improved measure to avoid that limitation and by now has been tested in different samples, showing strong psychometric properties in patients with chronic diseases [21].

Previous studies analysing BMI effects have pointed out that 30 Kg/m^2^ is the cut-off point to express significant inequalities on HRQL [14, 22, 23]. Accordingly, only the presence of obesity has significantly decreased HRQL. In the case of overweight individuals, there were no significant differences compared to those of normal weight [12, 19]. In our study, this feature was dependent on social class in the raw analysis, in which overweight was always associated with worse utility values, except for men in the high and middle class. Thus, the interaction with social class made apparent the protective effect of upper social class, triggering interest about its explanation. Different authors have discussed the mechanisms mediating the relationship between obesity and low HRQL. Possible mediators related to obesity, such as associated chronic conditions, have been postulated [12, 24]. We explored the role of the interaction between BMI and social class in modulating HRQL through regression analysis. Thus, we found that overweight and obesity consequences were not the same for individuals of high social class. In the low social class, the general rule was accomplished, and only obese individuals appeared to be associated with worse HRQL. In the middle class, on the contrary, the gap was set between overweight and normal-weight people. However, the most striking result was the finding that obese and overweight people of high social class have the same probability of the perfect health state as normal-weight individuals. In an attempt to explain that, we have analysed the association of chronic conditions, BMI categories and social class. But, the differences shed only partial light, as the percentage of upper class obese people with no chronic conditions in our sample was lower (45%) than the percentage of normal-weight individuals of high social class (67%), but higher than the percentage of obese people of lower class with no chronic conditions (35%). Besides physical health, Muenning et al. postulated a two-way relationship between obesity and impaired mental health by underscoring the causal role of stress, anxiety and depression [25].

The association between obesity and a low quality of life has been linked to the stigmatisation of obese individuals in society. This concept is understood as the possession of an attribute that is linked to a devalued social identity [26–28]. Various social groups have been found to describe obese individuals generically in very negative terms, such as lazy and unclean, with use of other similar stereotypes [27, 28]. The stigmatisation factor conditions the social and attitudinal environment in which obese people live and conduct their lives [24]. With other questionnaires, like Short-Form 36, this condition has been related to the negative impact on social function and mental health [24]. According to our results, it seems that the weight-based stigmatization experienced by overweight and obese people is present mainly in the low social class. This suggests that belonging to the upper social class could eliminate this environmental reaction to obesity and therefore, avoid the perception of unpleasant HRQL. In our results, the effects of the interactions between BMI and social class were consistent with the stigmatisation hypothesis. In fact, mobility limitation remained associated with obesity in the middle social class and associated with overweight in the high social class, underlining its effect on physical autonomy, whereas its effect on other dimensions, such as mental pain/discomfort or anxiety and depression, almost disappeared. Self-care limitation also disappeared, but this effect could be explained by a higher social support, as some differences were observed in usual activities for overweight people in the high social class. Puhl et al. have pointed out the detrimental effect of obesity-related stigma on health disparities and described it as a barrier for public health interventions [29].

The chronic conditions that decreased HRQL the most in both men and women were stroke and mental diseases. Their high impact on HRQL, measured by raw disutility values, was biased by the different distribution by age, gender and social class. When they were adjusted in the regression analysis, disutility values decreased considerably. It is clear that chronic conditions partially explain the lower HRQL of women. Systematically, the percentage of utility lost in women with one chronic condition is higher than utility lost in men with the same chronic condition. Stroke is associated in women with a reduction of 23%, while in men that reduction is 15%. Thus, women not only had more chronic conditions but also the presence of such conditions further decreased the HRQL. The perception of health is dependent on culture and environment [30]. In the Spanish population, Guallar-Castillón et al. pointed out that lifestyle may partially justify differences in HRQL between women and men [31]. In our results, the gender inequality in mental health is actually expressed in the “anxiety and depression” dimension of EQ-5D, showing the biggest loss in interaction between gender and BMI. Moreover, the higher prevalence of mental diseases may also explain the role of gender, in that mental health diseases are the second chronic determinant of disutility. In the analysis of chronic conditions, the disutility values could be assimilated to the disability weights for each disease calculated within the global burden of disease study carried out by the World Health Organization [32]. A disability weight reflects the severity of the disease on a scale from 0 (perfect health) to 1 (equivalent to death) and is used to calculate years lost due to disability (YLD) for the specific condition.

Certain limitations of this study should be mentioned. This is a cross-sectional study, which means that no cause-effect relationships can be established. Nonetheless, BMI was calculated from height and weight reported by the participants, which may not be accurate. Self-reported height and weight tend to be higher and lower, respectively, than the corresponding measured values. As a result, BMI values based on self-reported information may be underestimated. In addition, BMI values obtained from face-to-face interviews are widely used in research studies [12, 33, 34] and health surveys [11] as they are an easy and quick way to obtain such information. Similarly, self-reported diagnosis was used for chronic conditions; self-reports can result in under-reporting and also in the underestimating of the effect of chronic conditions on HRQL. In addition, coexistent diseases were not taken into account due to HRQL heterogeneity and a reduced number of cases for disease combinations.

Despite the above-mentioned limitations, this study shows several strengths. First, the data used in this study came from the latest Spanish National Health Survey performed in 2011–2012 and includes a large sample of adult individuals that is considered representative of the Spanish population. Second, the computer-assisted personal interview, which we used to collect standardized information, not only improved the quality of the collected data but also reduced the time interval necessary to adapt the final database for statistical analysis [35]. Third, to the best of our knowledge, this is the first study that has used the Spanish EQ-5D-5L value set to estimate the inequalities among individuals in the Spanish population [36].

## Conclusions

The joint analysis of gender, age, educational level, social class, body mass index and chronic diseases on health-related quality of life in the Spanish population revealed important inequalities in health. Social class acted as a modulator of the stigma associated with obesity. Chronic conditions producing loss of autonomy were related to the greatest reduction in health-related quality of life. This is the first study using the Spanish EQ-5D-5L value set to estimate utilities.

## Additional file

Additional file 1: **Table S1.** Mean utilities in men and women by social class and body mass index (BMI) using utilities for EQ-5D-5L. **Table S2.** Mean Utilities (SD) in men and women by age groups and BMI categories. **Table S3.** Mean Utilities (SD) in men and women by age groups and social class. **Table S4.** Distribution of the sample by BMI category, social class and number of chronic conditions diagnosed. **Table S5.** Two-step regression analysis to estimate mean utility values adjusted by age and diagnosis of each specific disease for the total population or by sex. **Table S6.** Two-step regression analysis to estimate mean utility values adjusted by age and diagnosis of each specific disease for the total population or by social class. (PDF 269 kb)

## Abbreviations

AMI: Acute Myocardial Infarction
BMI: Body Mass Index
CI: Confidence Interval
COPD: Chronic Obstructive Pulmonary Disease
HRQL: Health Related Quality of Life
OR: Odds Ratio
QALY: Quality Adjusted Life Years
SHS: Spanish Health Survey
YLD: Years Lost due to Disability

## References

[1] Marmot M (2005). Social determinants of health inequalities. Lancet Lond Engl.

[2] Arcaya MC, Arcaya AL, Subramanian SV (2015). Inequalities in health: definitions, concepts, and theories. Glob Health Action.

[3] EC PDL (1993). Health status and health policy: quality of life in health care evaluation and resource allocation.

[4] Khanna D, Tsevat J (2007). Health-related quality of life--an introduction. Am J Manag Care.

[5] Wisløff T, Hagen G, Hamidi V, Movik E (2014). Estimating QALY gains in applied studies: a review of cost-utility analyses published in 2010. PharmacoEconomics..

[6] EuroQol Group (1990). EuroQol--a new facility for the measurement of health-related quality of life. Health Policy.

[7] Herdman M, Gudex C, Lloyd A (2011). Development and preliminary testing of the new five-level version of EQ-5D (EQ-5D-5L). Qual Life Res.

[8] Rabin R, de Charro F (2001). EQ-5D: a measure of health status from the EuroQol group. Ann Med.

[9] Ramos-Goñi JM, Craig BM, Oppe M (2018). Handling data quality issues to estimate the Spanish EQ-5D-5L value set using a hybrid interval regression approach. Value Health.

[10] Hernandez Gimena, Garin Olatz, Pardo Yolanda, Vilagut Gemma, Pont Àngels, Suárez Mónica, Neira Montse, Rajmil Luís, Gorostiza Inigo, Ramallo-Fariña Yolanda, Cabases Juan, Alonso Jordi, Ferrer Montse (2018). Validity of the EQ–5D–5L and reference norms for the Spanish population. Quality of Life Research.

[11] Spanish National Health Survey 2011-2012. Spanish National Health Statistics 2017. [Internet]. [cited 2018 May 23]. Available from: http://www.ine.es/dyngs/INEbase/en/operacion.htm?c=Estadistica_C&cid=1254736176783&menu=resultados&secc=1254736195295&idp=1254735573175.

[12] Busutil R, Espallardo O, Torres A (2017). The impact of obesity on health-related quality of life in Spain. Health Qual Life Outcomes.

[13] Mar J, Larrañaga I, Arrospide A (2010). Impact of disability on different domains of health-related quality of life in the noninstitutionalized general population. Clin Outcomes Res CEOR.

[14] Lubetkin EI, Jia H, Franks P (2005). Relationship among sociodemographic factors, clinical conditions, and health-related quality of life: examining the EQ-5D in the U.S. general population. Qual Life Res.

[15] Oliva-Moreno J, Lopez-Bastida J, Worbes-Cerezo M (2010). Health related quality of life of Canary Island citizens. BMC Public Health.

[16] Domingo-Salvany A, Regidor E, Alonso J, Alvarez-Dardet C (2000). Proposal for a social class measure. Working Group of the Spanish Society of epidemiology and the Spanish Society of Family and Community Medicine. Aten Primaria.

[17] World Health Organization. Obesity: Preventing and Managing the Global Epidemic. World Health Organization; 2000. 267 p. 11234459

[18] Starkie HJ, Briggs AH, Chambers MG (2011). Predicting EQ-5D values using the SGRQ. Value Health.

[19] Martín-Fernández J, Ariza-Cardiel G, Plentinos-Castro E, et al. Explaining the difference in perceived health-related quality of life: a study within the Spanish population. Gac Sanit. 2018;32:447–53. 10.1016/j.gaceta.2017.05.016 28958573

[20] Marra CA, Woolcott JC, Kopec JA (1982). A comparison of generic, indirect utility measures (the HUI2, HUI3, SF-6D, and the EQ-5D) and disease-specific instruments (the RAQoL and the HAQ) in rheumatoid arthritis. Soc Sci Med.

[21] Sakthong P, Sonsa-Ardjit N, Sukarnjanaset P (2015). Psychometric properties of the EQ-5D-5L in Thai patients with chronic diseases. Qual Life Res.

[22] Serrano-Aguilar P, Muñoz-Navarro SR, Ramallo-Fariña Y (2009). Obesity and health related quality of life in the general adult population of the Canary Islands. Qual Life Res.

[23] Oliva-Moreno J, Gil-Lacruz A (2013). Body weight and health-related quality of life in Catalonia, Spain. Eur J Health Econ.

[24] Mar J, Karlsson J, Arrospide A (2013). Two-year changes in generic and obesity-specific quality of life after gastric bypass. Eat Weight Disord.

[25] Muennig P, Lubetkin E, Jia H, Franks P (2006). Gender and the burden of disease attributable to obesity. Am J Public Health.

[26] Ogden Jane, Clementi Cecelia (2010). The Experience of Being Obese and the Many Consequences of Stigma. Journal of Obesity.

[27] Puhl RM, Heuer CA (2009). The stigma of obesity: a review and update. Obes Silver Spring..

[28] Andreyeva T, Puhl RM, Brownell KD (2008). Changes in perceived weight discrimination among Americans, 1995-1996 through 2004-2006. Obes Silver Spring.

[29] Puhl RM, Heuer CA (2010). Obesity stigma: important considerations for public health. Am J Public Health.

[30] Bhui K, Dinos S (2008). Health beliefs culture. Dis Manag Heal Outcomes.

[31] Guallar-Castillón P, Redondo Sendino A, Banegas JR (2005). Differences in quality of life between women and men in the older population of Spain. Soc Sci Med.

[32] Lopez AD, Mathers CD, Ezzati M (2006). Global burden of disease and risk factors.

[33] Jia H, Lubetkin EI (2005). The impact of obesity on health-related quality-of-life in the general adult US population. J Public Health Oxf Engl.

[34] Xu Y, Zhou Z, Li Y (2015). Exploring the nonlinear relationship between body mass index and health-related quality of life among adults: a cross-sectional study in Shaanxi Province, China. Health Qual Life Outcomes.

[35] McColl E, Jacoby A, Thomas L (2001). Design and use of questionnaires: a review of best practice applicable to surveys of health service staff and patients. Health Technol Assess.

[36] Oppe M, Devlin NJ, van Hout B (2014). A program of methodological research to arrive at the new international EQ-5D-5L valuation protocol. Value Health.

