# Supplementary material for: Inequalities in health-related quality of life according to age, gender, educational level, social class, body mass index and chronic diseases using the Spanish value set for Euroquol 5D-5L questionnaire
Source: Health Qual Life Outcomes. 2019 Apr 18;17:69. doi: 10.1186/s12955-019-1134-9 (PMC6472013; doi:10.1186/s12955-019-1134-9)
Supplement: Supplementary file 1 — Table S1. Mean utilities in men and women by social class and body mass index (BMI) using utilities for EQ-5D-5L. Table S2. Mean Utilities (SD) in men and women by age groups and BMI categories. Table S3. Mean Utilities (SD) in men and women by age groups and social class. Table S4. Distribution of the sample by BMI category, social class and number of chronic conditions diagnosed. Table S5. Two-step regression analysis to estimate mean utility values adjusted by age and diagnosis of each specific disease for the total population or by sex. Table S6. Two-step regression analysis to estimate mean utility values adjusted by age and diagnosis of each specific disease for the total population or by social class. (PDF 269 kb) [file 12955_2019_1134_MOESM1_ESM.pdf]

SUPPLEMENTARY DATA

Table 1S. Mean utilities in men and women by social class and body mass index (BMI) using utilities for EQ-5D-5L.

Sex	Social class	BMI < 25 Mean (SD)	BMI 25-30 Mean (SD)	BMI ≥ 30 Mean (SD)	Total Mean (SD)
**Men**	Total	0.9567 (0.12)	0.9474 (0.13)*	0.9145 (0.17)*	0.9447 (0.14)
	Low	0.9470 (0.13)	0.9348 (0.14)*	0.9119 (0.16)*	0.9337 (0.14)
	Middle	0.9579 (0.13)	0.9536 (0.12)	0.9151 (0.18)*	0.9487 (0.14)
	High	0.9732 (0.08)	0.9651 (0.11)	0.9251 (0.19)*	0.9638 (0.12)
**Women**	Total	0.9333 (0.15)	0.8891 (0.18)*	0.8087 (0.25)*	0.8998 (0.18)
	Low	0.9165 (0.17)	0.8759 (0.19)*	0.7966 (0.26)*	0.8778 (0.20)
	Middle	0.9405 (0.14)	0.8993 (0.16)*	0.8138 (0.24)*	0.9111 (0.17)
	High	0.9518 (0.12)	0.9105 (0.17)*	0.8666 (0.22)*	0.9344 (0.15)
**Total**	Total	0.9424 (0.14)	0.9238 (0.15)*	0.8632 (0.22)*	0.9216 (0.16)
	Low	0.9285 (0.16)	0.9091 (0.17)*	0.8528 (0.22)*	0.9045 (0.18)
	Middle	0.9473 (0.14)	0.9324 (0.14)*	0.8702 (0.21)*	0.9298 (0.15)
	High	0.9597 (0.11)	0.9462 (0.14)*	0.8989 (0.21)*	0.9487 (0.13)

BMI: Body Mass Index; SD: standard deviation; *Statistically significant differences in relation to the mean of BMI group <25

TABLE 2S. Mean Utilities (SD) in men and women by age groups and BMI categories.

Sex	Age	BMI < 25	BMI 25-30	BMI≥ 30	Total
**Men**	Total	0.9567 (0.12)	0.9474 (0.13)*	0.9145 (0.17)*	0.9447 (0.14)
	< 30	0.9856 (0.07)	0.9823 (0.06)	0.9652 (0.13)	0.9834 (0.07)
	30-39	0.9778 (0.09)	0.9746 (0.10)	0.9668 (0.08)	0.9748 (0.10)
	40-49	0.9619 (0.11)	0.9670 (0.09)	0.9563 (0.10)	0.9634 (0.10)
	50-59	0.9396 (0.14)	0.9542 (0.10)	0.9102 (0.18)	0.9395 (0.14)
	60-69	0.9331 (0.16)	0.9454 (0.12)	0.9092 (0.16)	0.9332 (0.14)
	70-79	0.9212 (0.14)	0.8990 (0.18)	0.8537 (0.21)	0.8935 (0.18)
	80-89	0.8305 (0.22)	0.8078 (0.26)	0.7351 (0.30)	0.8010 (0.25)
	>89	0.7040 (0.26)	0.7003 (0.27)	0.2056 (0.00)	0.6882 (0.27)
**Women**	Total	0.9333 (0.15)	0.8891 (0.18)*	0.8087 (0.25)*	0.8998 (0.18)
	< 30	0.9827 (0.06)	0.9826 (0.05)	0.9461 (0.17)	0.9805 (0.07)
	30-39	0.9709 (0.07)	0.9650 (0.07)	0.9356 (0.14)	0.9661 (0.08)
	40-49	0.9510 (0.11)	0.9460 (0.11)	0.9206 (0.15)	0.9459 (0.12)
	50-59	0.9124 (0.18)	0.9003 (0.15)	0.8439 (0.21)	0.8951 (0.18)
	60-69	0.9154 (0.16)	0.8674 (0.18)	0.7965 (0.22)	0.8696 (0.19)
	70-79	0.8532 (0.18)	0.8520 (0.18)	0.7218 (0.28)	0.8155 (0.22)
	80-89	0.7529 (0.28)	0.7311 (0.28)	0.6007 (0.31)	0.7078 (0.29)
	>89	0.5547 (0.32)	0.5593 (0.36)	0.4246 (0.34)	0.5397 (0.34)
**Total**	Total	0.9424 (0.14)	0.9238 (0.15)*	0.8632 (0.22)*	0.9216 (0.16)
	< 30	0.9840 (0.07)	0.9824 (0.05)	0.9564 (0.15)	0.9820 (0.07)
	30-39	0.9735 (0.08)	0.9714 (0.09)	0.9543 (0.11)	0.9705 (0.09)
	40-49	0.9548 (0.11)	0.9599 (0.10)	0.9418 (0.13)	0.9548 (0.11)
	50-59	0.9219 (0.17)	0.9322 (0.12)	0.8810 (0.20)	0.9172 (0.16)
	60-69	0.9218 (0.16)	0.9107 (0.15)	0.8548 (0.20)	0.9004 (0.17)
	70-79	0.8775 (0.17)	0.8751 (0.18)	0.7708 (0.26)	0.8487 (0.21)
	80-89	0.7838 (0.26)	0.7665 (0.27)	0.6510 (0.31)	0.7469 (0.28)
	>89	0.5910 (0.31)	0.6053 (0.33)	0.4090 (0.33)	0.5773 (0.33)

TABLE 3S. Mean Utilities (SD) in men and women by age groups and social class.

Sex	Age	Low social class	Middle social class	High social class	Total
**Men**	Total	0.9337 (0.14)	0.9487 (0.14)	0.9638 (0.12)	0.9447 (0.14)
	< 30	0.9815 (0.07)	0.9841 (0.09)	0.9872 (0.05)	0.9834 (0.07)
	30-39	0.9685 (0.11)	0.9790 (0.08)	0.9799 (0.09)	0.9748 (0.10)
	40-49	0.9573 (0.11)	0.9643 (0.10)	0.9745 (0.08)	0.9634 (0.10)
	50-59	0.9246 (0.14)	0.9430 (0.14)	0.9637 (0.12)	0.9395 (0.14)
	60-69	0.9245 (0.14)	0.9323 (0.15)	0.9564 (0.12)	0.9332 (0.14)
	70-79	0.8767 (0.18)	0.9056 (0.18)	0.9300 (0.16)	0.8935 (0.18)
	80-89	0.8008 (0.26)	0.7930 (0.26)	0.8203 (0.25)	0.8010 (0.25)
	>89	0.6728 (0.27)	0.7141 (0.25)	0.6979 (0.37)	0.6882 (0.27)
**Women**	Total	0.8778 (0.20)	0.9111 (0.17)	0.9344 (0.15)	0.8998 (0.18)
	< 30	0.9775 (0.07)	0.9865 (0.05)	0.9786 (0.10)	0.9805 (0.07)
	30-39	0.9584 (0.09)	0.9712 (0.08)	0.9726 (0.06)	0.9661 (0.08)
	40-49	0.9357 (0.13)	0.9440 (0.11)	0.9671 (0.08)	0.9459 (0.12)
	50-59	0.8718 (0.19)	0.9026 (0.18)	0.9341 (0.13)	0.8951 (0.18)
	60-69	0.8383 (0.21)	0.8809 (0.18)	0.9254 (0.11)	0.8696 (0.19)
	70-79	0.7970 (0.24)	0.8312 (0.20)	0.8638 (0.18)	0.8155 (0.22)
	80-89	0.6814 (0.29)	0.7562 (0.26)	0.7128 (0.33)	0.7078 (0.29)
	>89	0.5725 (0.34)	0.5657 (0.35)	0.3295 (0.21)	0.5397 (0.34)
**Total**	Total	0.9045 (0.18)	0.9298 (0.16)	0.9487 (0.13)	0.9216 (0.16)
	< 30	0.9795 (0.07)	0.9853 (0.07)	0.9830 (0.08)	0.9820 (0.07)
	30-39	0.9636 (0.10)	0.9752 (0.08)	0.9761 (0.08)	0.9705 (0.09)
	40-49	0.9468 (0.12)	0.9545 (0.11)	0.9707 (0.08)	0.9548 (0.11)
	50-59	0.8971 (0.17)	0.9234 (0.16)	0.9489 (0.13)	0.9172 (0.16)
	60-69	0.8799 (0.19)	0.9062 (0.17)	0.9403 (0.12)	0.9004 (0.17)
	70-79	0.8297 (0.22)	0.8636 (0.20)	0.8948 (0.17)	0.8487 (0.21)
	80-89	0.7312 (0.28)	0.7720 (0.26)	0.7571 (0.30)	0.7469 (0.28)
	>89	0.5986 (0.32)	0.6002 (0.33)	0.4318 (0.31)	0.5773 (0.33)

TABLE 4S. Distribution of the sample by BMI category, social class and number of chronic conditions diagnosed.

	**Low social class**	**Middle social class**	**High social class**	p-valor
**BMI < 25**				
**Number of chronic conditions diagnosed**				
None	2,036 (59.0%)	1,835 (63.0%)	1,311 (67.4%)	
One or two	929 (26.9%)	773 (26.6%)	486 (25.0%)	
More than two	485 (14.1%)	304 (10.4%)	148 (7.6%)	< 0.001
**BMI 25-30**				
**Number of chronic conditions diagnosed**				
None	1,514 (46.1%)	1,274 (52.6%)	706 (57.2%)	
One or two	1,021 (31.1%)	735 (30.4%)	367 (29.7%)	
More than two	752 (22.8%)	411 (17.0%)	162 (13.1%)	< 0.001
**BMI≥ 30**				
**Number of chronic conditions diagnosed**				
None	654 (34.7%)	364 (38.2%)	165 (45.1%)	
One or two	583 (31.0%)	310 (32.6%)	117 (32.0%)	
More than two	646 (34.3%)	278 (29.2%)	84 (22.9%)	< 0.001
**Total**				
**Number of chronic conditions diagnosed**				
None	4,204 (48.8%)	3,473 (55.3%)	2,182 (61.5%)	
One or two	2,533 (29.4%)	1,818 (28.9%)	970 (27.4%)	
More than two	1,883 (21.8%)	993 (15.8%)	394 (11.1%)	< 0.001

TABLE 5S. Two-step regression analysis to estimate mean utility values adjusted by age and diagnosis of each specific disease for the total population or by sex.

		Constant	Age	Diagnosis	Sex	Sex*Diagnosis
**Total population**						
Acute myocardial infarction	Step 1	0.7416	-0.0457	-0.6620		
	Step 2	-1.6225	0.0188	0.2057		
Other heart diseases	Step 1	0.7833	-0.0434	-0.9131		
	Step 2	-1.6374	0.0176	0.2440		
Arthrosis, arthritis or rheumatism	Step 1	1.3818	-0.0341	-1.6506		
	Step 2	-1.7740	0.0176	0.2545		
Chronic obstructive pulmonary disease	Step 1	0.7798	-0.0449	-1.0251		
	Step 2	-1.6354	0.0186	0.2179		
Diabetes	Step 1	0.7777	-0.0441	-0.6277		
	Step 2	-1.6431	0.0181	0.2304		
Cirrhosis, hepatic dysfunction	Step 1	0.7415	-0.0463	-1.0872		
	Step 2	-1.6266	0.0191	0.4053		
Mental health diseases	Step 1	1.0298	-0.0448	-2.3454		
	Step 2	-1.7669	0.0194	0.4312		
Stroke	Step 1	0.7454	-0.0457	-1.4934		
	Step 2	-1.6316	0.0183	0.5691		
**Population by sex**						
Acute myocardial infarction	Step 1	1.0873	-0.0453	-0.7134	-0.6383	-0.7915
	Step 2	-1.6996	0.0186	0.1591	0.1240	0.2095
Other heart diseases	Step 1	1.1317	-0.0431	-0.9750	-0.6448	0.0197
	Step 2	-1.6969	0.0173	0.1498	0.0983	0.1665
Arthrosis, arthritis or rheumatism	Step 1	1.5376	-0.0343	-1.4855	-0.3343	-0.1908
	Step 2	-1.7536	0.0175	0.1404	-0.0391	0.1769
Chronic obstructive pulmonary disease	Step 1	1.1230	-0.0447	-1.0101	-0.6354	-0.1339
	Step 2	-1.7094	0.0184	0.1710	0.1180	0.1079
Diabetes	Step 1	1.1236	-0.0437	-0.6492	-0.6371	-0.0972
	Step 2	-1.7097	0.0178	0.1697	0.1086	0.1161
Cirrhosis, hepatic dysfunction	Step 1	1.0782	-0.0461	-1.1140	-0.6284	-0.1117
	Step 2	-1.7071	0.0189	0.5006	0.1301	-0.1506
Mental health diseases	Step 1	1.2663	-0.0448	-2.3730	-0.4673	0.1592
	Step 2	-1.8047	0.0193	0.4550	0.0669	-0.0466
Stroke	Step 1	1.0877	-0.0455	-1.6303	-0.6374	0.1133
	Step 2	-1.7119	0.0180	0.5232	0.1305	0.1230

Step 1: Logistic regression; Step 2: Generalized linear regression (family: gamma, link: logarithmic).

TABLE 6S. Two-step regression analysis to estimate mean utility values adjusted by age and diagnosis of each specific disease for the total population or by social class.

		Constant	Age	Middle social class	High social class	Diagnosis	Middle social class *Diagnosis	High social class *Diagnosis
**Population by social class**								
Acute myocardial infarction	Step 1	0.5408	-0.0453	0.2839	0.5652	-0.9742	0.4810	1.0271
	Step 2	-1.6379	0.0158	-0.0776	-0.1348	0.1492	0.0454	0.2843
Other heart diseases	Step 1	0.5827	-0.0430	0.2783	0.5707	-0.9730	0.1933	0.0777
	Step 2	-1.6490	0.0146	-0.0814	-0.1555	0.2009	0.0424	0.1928
Arthrosis, arthritis or rheumatism	Step 1	1.2066	-0.0338	0.2622	0.4249	-1.6447	-0.0331	0.1398
	Step 2	-1.8371	0.0144	0.0233	-0.0823	0.3101	-0.1265	-0.0279
Chronic obstructive pulmonary disease	Step 1	0.5842	-0.0445	0.2698	0.5532	-1.0693	0.1826	0.2345
	Step 2	-1.6599	0.0156	-0.0626	-0.1147	0.2369	-0.0492	0.0196
Diabetes	Step 1	0.5852	-0.0438	0.2673	0.5335	-0.7038	0.1872	0.4328
	Step 2	-1.6655	0.0150	-0.0606	-0.1181	0.2223	-0.0387	0.0987
Cirrhosis, hepatic dysfunction	Step 1	0.5333	-0.0458	0.2953	0.5873	-1.1531	-0.0050	0.3149
	Step 2	-1.6437	0.0160	-0.0801	-0.1216	0.3875	0.1104	-0.1213
Mental health diseases	Step 1	0.8494	-0.0444	0.2276	0.5452	-2.3496	0.2270	-0.3049
	Step 2	-1.8440	0.0163	-0.0229	-0.0743	0.5486	-0.0742	-0.0671
Stroke	Step 1	0.5334	-0.0452	0.3010	0.5991	-1.2226	-0.5603	-0.5274
	Step 2	-1.6423	0.0154	-0.0865	-0.1399	0.4498	0.1468	0.0701

Step 1: Logistic regression; Step 2: Generalized linear regression (family: gamma, link: logarithmic).
